# The phosphatidylinositol 3-phosphate-binding protein SNX4 controls ATG9A recycling and autophagy

**DOI:** 10.1242/jcs.250670

**Published:** 2021-02-10

**Authors:** Anthony Ravussin, Andreas Brech, Sharon A. Tooze, Harald Stenmark

**Affiliations:** 1 Centre for Cancer Cell Reprogramming, Faculty of Medicine, University of Oslo, Montebello, 0379 Oslo, Norway; 2 Department of Molecular Cell Biology, Institute for Cancer Research, Oslo University Hospital, Montebello, 0379 Oslo, Norway; 3 Molecular Cell Biology of Autophagy Laboratory, The Francis Crick Institute, London NW1 1AT, UK

**Keywords:** Autophagy, Endosome, Phosphoinositide, Recycling

## Abstract

Late endosomes and lysosomes (endolysosomes) receive proteins and cargo from the secretory, endocytic and autophagic pathways. Although these pathways and the degradative processes of endolysosomes are well characterized, less is understood about protein traffic from these organelles. In this study, we demonstrate the direct involvement of the phosphatidylinositol 3-phosphate (PI3P)-binding SNX4 protein in membrane protein recycling from endolysosomes, and show that SNX4 is required for proper autophagic flux. We show that SNX4 mediates recycling of the lipid scramblase ATG9A, which drives expansion of nascent autophagosome membranes, from endolysosomes to early endosomes, from where ATG9A is recycled to the trans-Golgi network in a retromer-dependent manner. Upon siRNA-mediated depletion of SNX4 or the retromer component VPS35, we observed accumulation of ATG9A on endolysosomes and early endosomes, respectively. Moreover, starvation-induced autophagosome biogenesis and autophagic flux were inhibited when SNX4 was downregulated. We propose that proper ATG9A recycling by SNX4 sustains autophagy by preventing exhaustion of the available ATG9A pool.

This article has an associated First Person interview with the first author of the paper.

## Abstract (precis)

**Highlighted Article:** The PI3P-binding protein SNX4 mediates recycling of the autophagy-promoting lipid scramblase ATG9A from lysosomes and autolysosomes, allowing reuse of this enzyme for rounds of autophagosome biogenesis.

## INTRODUCTION

Macroautophagy (hereafter referred to as autophagy) controls numerous fundamental physiological functions (Levine and Klionsky, 2004; Mizushima, 2005; Ohsumi, 2001). During the autophagic process, sequestration of cytoplasmic material ensues within double membrane autophagosomes, which fuse with lysosomes for degradation of contents in the resulting autolysosomes (Klionsky, 2005; Levine and Klionsky, 2004; Nakatogawa et al., 2009). Conserved autophagy-related gene (ATG) proteins act in a concerted manner in order to regulate proper autophagosome biogenesis and autophagic flux (Mizushima and Levine, 2010). Strict temporal and spatial regulation of the recruitment of membrane proteins and ATG proteins is required for proper autophagic progression and flux. This is in part achieved by specific membrane lipids and association of lipid-binding proteins, in particular phosphatidylinositol 3-phosphate (PI3P). PI3P is mainly generated by the class III phosphatidylinositol 3-kinase VPS34 through phosphorylation of phosphatidylinositol and has been shown to be essential during the early phase of phagophore biogenesis via recruitment of its effectors DFCP1 (also known as ZFYVE1) and WIPI2 (Axe et al., 2008; Dooley et al., 2015). These proteins are required in order to recruit the only membrane-spanning ATG protein, ATG9A, to the pre-autophagosomal structure and forming phagophore (Funderburk et al., 2010; Takahashi et al., 2011). ATG9A and its yeast counterpart were recently shown to have lipid scramblase activity, thereby translocating phospholipids between the two monolayers of lipid bilayers, presumably in order to drive expansion of the nascent autophagosome membrane (Maeda et al., 2020; Matoba et al., 2020).

ATG9A cycles primarily between the Golgi and endosomes in mammalian cells (Young et al., 2006), with small amounts found on the plasma membrane (Ravikumar et al., 2010). The known residence of ATG9A in endosomal compartments includes EEA1-positive early endosomes (Puri et al., 2013), Rab7-positive late endosomes (Young et al., 2006) and Rab11-positive recycling endosomes (Knaevelsrud et al., 2013; Lamb et al., 2016; Longatti et al., 2012). ATG9A vesicles are essential for autophagosome formation, and studies in yeast have shown that the vast majority of Atg9 vesicles are derived from the Golgi apparatus in a process involving Atg23 and Atg27, and that these vesicles assemble individually into the pre-autophagosomal structure upon starvation-induced autophagy (Yamamoto et al., 2012). In mammalian cells, whereas the majority of LC3-positive autophagsomes do not contain ATG9A, there may be small amounts of ATG9A mislocalized to the membrane of the autophagosome, or indeed arriving from the plasma membrane through late endosomes, which end up on the limiting membrane of the endolysosome. Although the recruitment and retrieval of ATG9A to and from sites of the forming phagophore is becoming better understood (Orsi et al., 2012; Young et al., 2006), there are still unexplored questions about its trafficking in the endolysosomal system and upon termination of autophagy.

There are multiple PI3P-binding proteins other than DFCP1 and WIPI2 expressed in cells, raising the possibility that additional PI3P effectors could be involved in regulation of autophagy. The largest family of PI3P-binding proteins is the sorting nexin (SNX) family, a group of membrane-associated proteins containing a phox homology (PX) domain, most of which have been found to bind PI3P (Worby and Dixon, 2002). Of the 33 annotated human SNX proteins, a subfamily of SNXs contain a C-terminal BAR domain, and studies of these have shed light on a process of tubular-based endosomal sorting (Knaevelsrud et al., 2013). The SNX–BAR proteins participate in evolutionarily conserved protein complexes that coordinate membrane deformation within the concave surface of the dimerized BAR motif, which allows association with the phospholipid bilayer through electrostatic interactions, possibly for cargo selection (McMahon and Gallop, 2005; Peter et al., 2004; Wang et al., 2018).

In this study, we reveal the involvement of a PI3P binding SNX–BAR protein, SNX4, in ATG9A trafficking and autophagy. We find that SNX4 mediates recycling of ATG9A from endolysosomes and autolysosomes to early endosomes, and that it is essential for proper autophagy.

## RESULTS

### SNX4 localizes to LAMP1-positive endolysosomes and EEA1-positive early endosomes in a PI3P-dependent manner

**Fig. 1.**
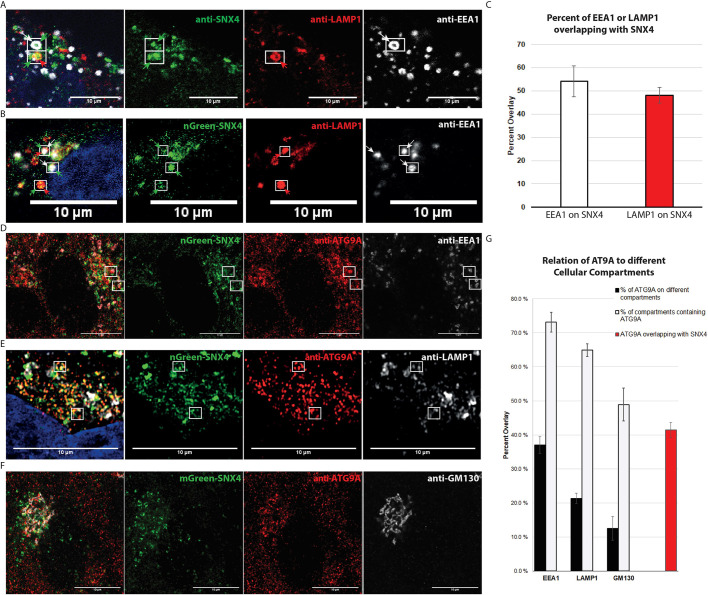
**SNX4 colocalizes with both early-endosomal and endolysosomal structures, and localizes together with ATG9A on different cellular compartments.** (A) Endogenous SNX4 (green) colocalizes with both Lamp1 (red) and EEA1 (greyscale). (B) Stably expressed mNeonGreen–SNX4 (nGreen–SNX4, green) colocalizes with both LAMP1 (red) and EEA1 (greyscale). (C) Manders’ Overlap quantification of overlap between tagged nGreen-SNX4 and EEA1 and LAMP1 signals. *n*=3 experiments. Mean±s.e.m. (D) Representative immunofluorescence images showing ATG9A (red) overlap with both SNX4 (nGreen–SNX4, green) and EEA1 (greyscale). (E) Representative immunofluorescence images showing ATG9A (red) overlap with both SNX4 (nGreen–SNX4, green) and LAMP1 (greyscale). (F) Representative immunofluorescence images showing ATG9A (red) overlap with both SNX4 (mGreen–SNX4, green) and GM130 (greyscale). (G) Manders’ Overlap quantification of overlap between cellular compartments and ATG9A, as well as SNX4 and ATG9A. Graph shows percentage of total ATG9A signal that overlaps with EEA1-, LAMP1- and GM130-labelled compartments (black), percentage of EEA1-, LAMP1- and GM130-labelled compartments that are also ATG9A positive (white), and the percentage of the total ATG9A signal that overlaps with SNX4 signal (red). Mean±s.e.m. of *n*=3 or 4 experiments, with a minimum of four images per experiment. Left-hand panels in A,B,D–F show merge images, with DNA stained using Hoechst (blue; A,B,E). Boxes in A,B,D,E show examples of colocalization between different channels. Arrows in A and B indicate by colour which proteins colocalize. Scale bars: 10 μm.

To determine the localization of SNX4 within the cell, we used an anti-SNX4 antibody for detection of endogenous protein and generated a stable retinal pigment epithelial cell line (RPE-1) expressing mNeonGreen–SNX4 for live imaging. Live-cell imaging and fixed-cell microscopy showed that SNX4 resides on both LAMP1-positive late endosomes/lysosomes (endolysosomes) (54.1%±6.7, mean±s.e.m.) and EEA1-positive early endosomes (48.1%±3.4) (Fig. 1A–C).

SNX4 has been reported to bind PI3P (Leprince et al., 2003) and is implicated in endosomal sorting. We investigated whether PI3P binding is required for recruitment of SNX4 to endosomes and endolysosomes. For this purpose, we incubated cells with SAR405, a highly specific inhibitor of VPS34, which led to rapid loss of SNX4 from endosomes (Movie 1). This shows that SNX4 is recruited to membranes in a PI3P dependent manner.

### ATG9A localizes to Golgi, endolysosomes and early endosomes, and partially colocalizes with SNX4

Next, we wanted to determine the relationship between SNX4 and ATG9A. To determine the localization of ATG9A within the cell, we created a double-tagged stable RPE-1 cell line expressing mNeonGreen–SNX4 and mCherry–ATG9A. Live-cell imaging showed that many of the SNX4-containing vesicles co-trafficked with ATG9A (Movie 2). Using endogenous antibody staining for ATG9A, we determined that 22.7%±6.9 (mean±s.e.m.) of SNX4-containing vesicles were decorated with ATG9A. Conversely, 41.4%±2.3 of ATG9A staining colocalized with SNX4 (Fig. 1G). Additionally, in wild-type RPE-1 cells in full medium, we observed a prominent juxtanuclear ATG9A staining that colocalized with the Golgi marker GM-130 (Fig. 1F). Quantification of colocalization revealed that 12.5%±3.5 of ATG9A colocalized with GM130 and 48.9%±4.9 of Golgi staining contained ATG9A staining. Moreover, 37.0%±2.4 of ATG9A colocalized with EEA1-positive early endosomes (Fig. 1D) and 21.4%±1.5 of ATG9A colocalized with LAMP1-positive endolysosomes (Fig. 1E). These data confirm that ATG9A is found on both Golgi and endosome membranes, as demonstrated previously (Imai et al., 2016), and additionally show a significant pool of ATG9A on endolysosomes.

### Inhibition of SNX4 increases the ATG9A localization on endolysosomes upon starvation-induced autophagy

**Fig. 2.**
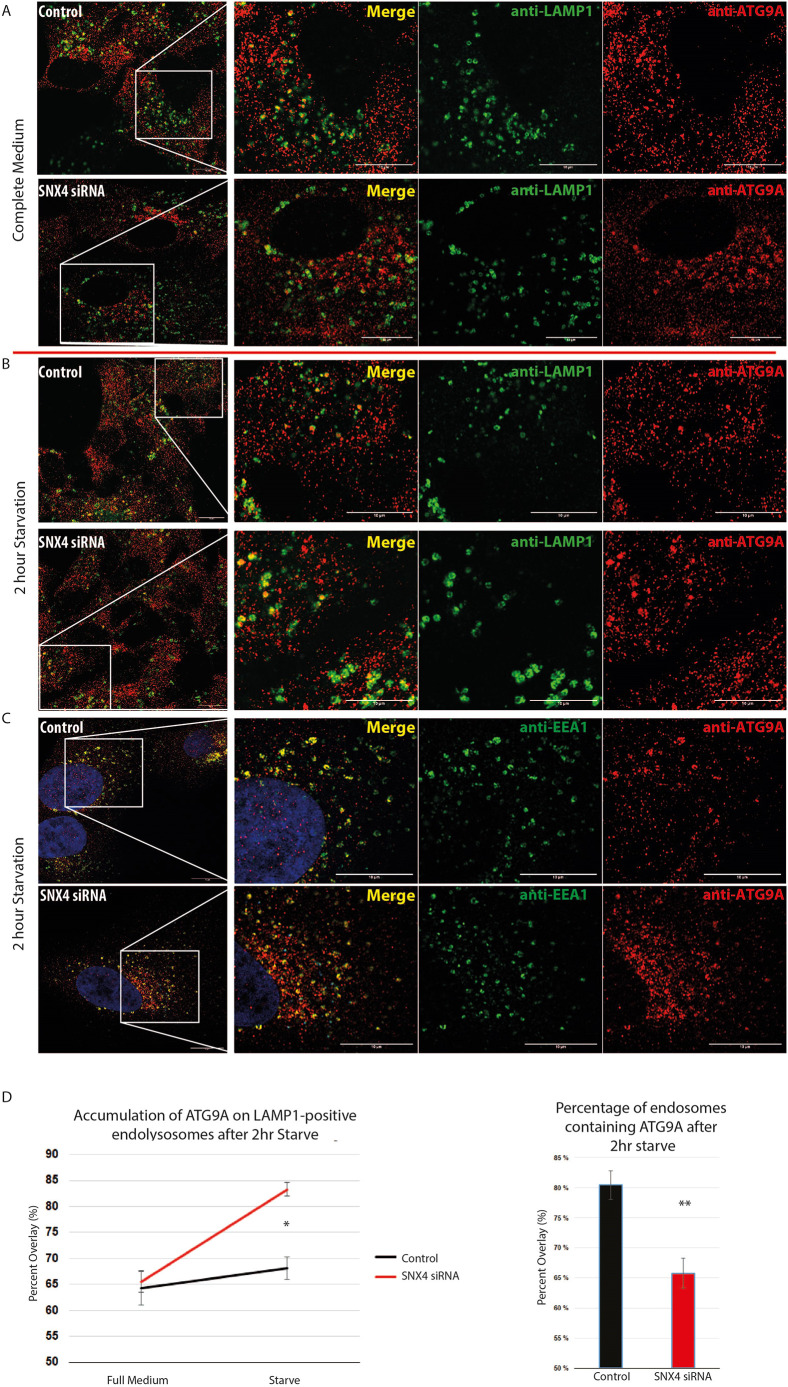
**ATG9A increases on LAMP1-positive structures and decreases on EEA1-positive structures during autophagy in SNX4-depleted cells.** (A) Representative immunofluorescence images showing ATG9A (red) overlapping with LAMP1 (green) in complete medium. (B) Representative immunofluorescence images showing ATG9A overlapping with LAMP1 after 2 h starvation. (C) Representative immunofluorescence images showing ATG9A (red) overlapping with EEA1 (green) after 2 h starvation. In A–C, cells were treated with SNX4 siRNA or scrambled siRNA (control) as indicated. Boxes highlight regions shown magnified in the right-hand panels. (D) In starved conditions, ATG9A increases colocalization with LAMP1-positive structures upon SNX4 depletion (left), and decreases on EEA1-positive early endosomes (right). Manders’ Overlap quantifications. Mean±s.e.m. of *n*>3 experiments (*P<0.01, **P<0.001). Scale bars: 10 μm.

As shown in Fig. 1, in full medium under basal conditions, ATG9A was found to be mainly juxtanuclear and to colocalize quite strongly with the Golgi complex. It is thought that this juxtanuclear ATG9A pool traffics through endosomes for fast mobility upon autophagy-inducing stress (Longatti et al., 2012; Young et al., 2006). In accordance with these previous studies, we found that in full medium, ATG9A in wild-type RPE-1 cells indeed localized to a juxtanuclear region (Fig. 1D–F, Fig. 2A). In full medium, siRNA-mediated knockdown of SNX4 did not seem to change the localization of ATG9A, although it slightly increased the perinuclear ATG9A intensity. In fact, the proportion of LAMP1 vesicles containing ATG9A in control cells compared to that in SNX4-knockdown cells was no different (64.3%±3.3 and 65.5%±2.0, control and SNX4 siRNA, respectively; mean±s.e.m.) (Fig. 2A,D).

Next, we assessed whether ATG9A localization changed upon inhibition of SNX4 when autophagy was induced by amino acid starvation. Strikingly, upon SNX4 knockdown, ATG9A was redistributed to a more peripheral localization (Fig. 2B). Consistent with previous studies, starvation-induced autophagy in wild-type RPE-1 cells also triggered dispersal of the ATG9A Golgi pool into a more peripheral localization. Interestingly, upon starvation, ATG9A did not seem to disperse and had a similar juxtanuclear localization in SNX4 siRNA-treated cells when compared to the localization in controls (Fig. 2B). Upon quantification, ATG9A was found to accumulate in LAMP1-positive endolysosomes in SNX4 siRNA inhibited cells upon starvation (Fig. 2D), potentially leading to larger LAMP1 vesicles (Fig. 2B). The percentage of LAMP1-positive vesicles containing ATG9A increased in SNX4 siRNA-treated cells to 83.2%±1.3 (Fig. 2D). Furthermore, ATG9A co-localization with the early-endosomal marker EEA1 decreased in SNX4 siRNA-treated cells (Fig. 2C,D). These data suggest that SNX4 is required for precise mobilization of the ATG9A protein during autophagy.

### Inhibition of retromer increases the localization of ATG9A on early endosomes

**Fig. 3.**
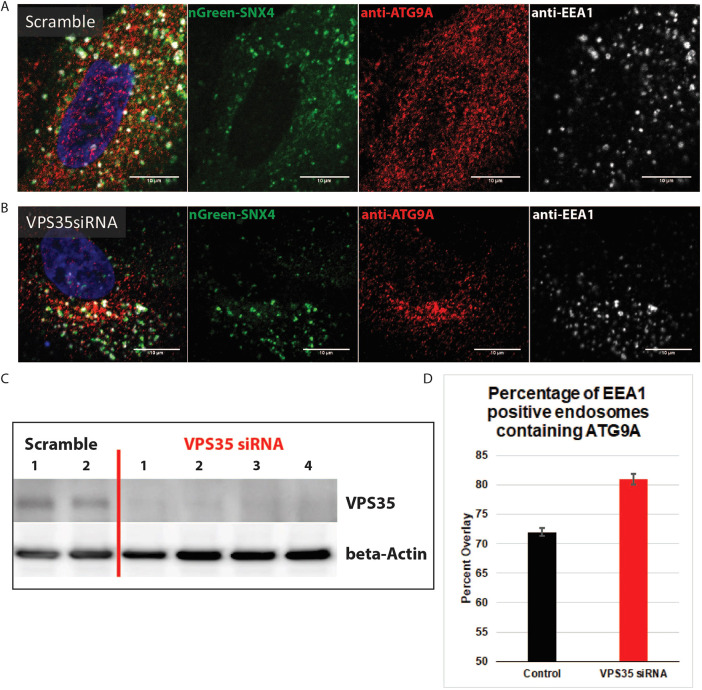
**ATG9A increases on early endosomes during autophagy when VPS35 is depleted.** (A) Representative immunofluorescence images showing ATG9A (red) overlapping with both mNeonGreen–SNX4 (nGreen–SNX4, green) and EEA1 (greyscale) in scramble control RPE-1 cells. (B) Representative immunofluorescence images showing ATG9A overlapping with both mNeonGreen–SNX4 and EEA1 in VPS35-depleted RPE-1 cells. In A and B, left-hand panel shows a merge image, with DNA stained using Hoechst (blue). Scale bars: 10 μm. (C) Western blot showing VPS35 knockdown efficiency in lysates from VPS35 siRNA-treated cells, compared with lysates from scrambled siRNA-treated cells. β-actin is shown as a loading control. Numbers indicate different knockdown cell populations with the same siRNA. (D) Manders’ Overlap quantification between ATG9A and EEA1 in control and VPS35-depleted cells. *n*=4 experiments. Mean±s.e.m. *P*<0.001.

VPS35 is the core functional component of retromer complex variants that act to ensure proper sorting of selected transmembrane cargo proteins from endosomes to the biosynthetic pathway. VPS35 is recruited to endosomal membranes and, with other SNX–BAR retromer subunits, mediates retrograde transport of cargo proteins from endosomes to the trans-Golgi network (Zavodszky et al., 2014). Previous studies have shown no effect of VPS26 retromer subunit in ATG9A redistribution or in the reestablishment of ATG9A juxtanuclear population (Orsi et al., 2012). To investigate whether ATG9A is recycled back to the Golgi via retromer complex in RPE-1 cells, we used siRNA inhibition of VPS35 to determine the fate of ATG9A. Interestingly, upon VPS35 knockdown (Fig. 3C), we observed that ATG9A had a stronger juxtanuclear localization (Fig. 3A,B). However, the peripheral ATG9A in these VPS35-depleted cells showed an increased colocalization with the early-endosomal marker EEA1 (Fig. 3D). The localization of ATG9A to EEA1-positive endosomes increased from 71.9%±0.7 (mean±s.e.m.) in control to 80.9%±1 in VPS35-depleted cells (Fig. 3D). This confirms the importance of retromer in recycling of ATG9A from endosomes to the Golgi.

### SNX4 is required for formation of LC3-positive autophagosomes upon starvation

**Fig. 4.**
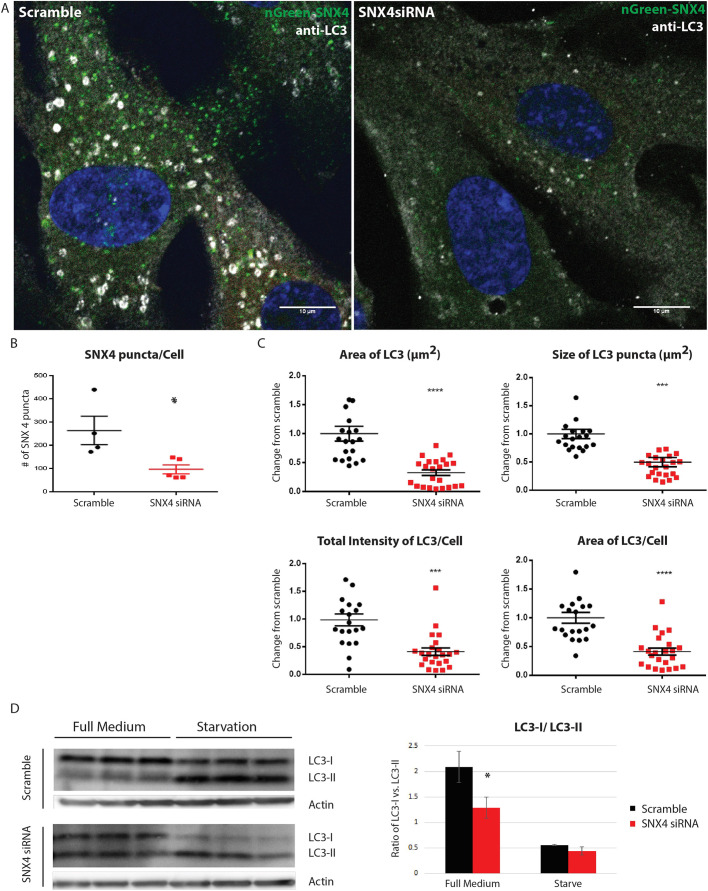
**Depletion of SNX4 inhibits starvation-induced formation of LC3-positive autophagosomes.** (A) Representative immunofluorescence micrograph showing LC3 (greyscale) upon starvation in scrambled siRNA-treated (scramble, left) and SNX4-depleted (right) RPE-1 cells. mNeonGreen–SNX4 (nGreen–SNX4) is shown in green, Hoechst staining of DNA is shown in blue. (B) Quantification of SNX4 puncta in scrambled siRNA-treated and SNX4-depleted RPE-1 cells. Mean±s.e.m. of four experiments. *P<0.05 (Student's *t*-test). (C) Quantification of total area of LC3 quantified per image (top left), average area of LC3 per cell (area of LC3 quantified divided by number of cells in frame of view; bottom right), average size of LC3 puncta (the area quantified of LC3 puncta divided by the number of individual puncta; top right) and total LC3 intensity/cell (bottom left) in RPE-1 cells treated with scrambled siRNA and SNX4 siRNA. All quantifications are from four independent experiments with 4–6 technical replicates each and are normalized to the mean value of the scramble control cells. There were 171 cells analysed for scrambled controls and 141 cells analysed for SNX4 siRNA samples. Mean±s.e.m. ***P<0.001; ****P<0.0001 (Student's *t*-test). (D) Western blot analysis of LC3-I and LC3-II in RPE-1 cells treated with scrambled siRNA or siRNA against SNX4, incubated in either full medium or for two hours under starvation conditions. Quantification of the ratio of LC3-I versus LC3-II normalized to actin loading controls. Note that the lower blot is shown at relatively higher exposure in order to facilitate the relative comparison of LC3-I versus LC3-II. Mean±s.e.m. of three experiments. *P=0.049 (Student's *t*-test).

To examine whether SNX4 is necessary for proper autophagy in mammalian cells, we tested the necessity of SNX4 during amino acid starvation-induced autophagy. During autophagy, the cytosolic form of microtubule-associated protein 1A/1B-light chain 3 (LC3, here referring to both MAP1LC3A and MAP1LC3B), LC3-I, is conjugated to phosphatidylethanolamine (PE) to form LC3-PE conjugate (LC3-II), which is recruited to autophagosomal membranes. Using an imaging-based approach, we observed that starvation-induced LC3 structures were significantly decreased upon inhibition of SNX4 (Fig. 4A,B). Quantitative image analyses showed that there were decreases in average LC3 area, average size of LC3 puncta and total LC3 intensity per cell (Fig. 4C). We next investigated whether there were changes in LC3 lipidation levels, which can be detected by western blotting (Klionsky et al., 2016). Interestingly, in full medium, SNX4-depleted cells had a higher proportion of the lipidated form (LC3-II) relative to the unlipidated form (LC3-I) compared with the levels in control cells (Fig. 4D). This suggests that there is a higher level of baseline LC3-II lipidation in SNX4-depleted cells. This could be due to decreased turnover of LC3-II (see below).

### Depletion of SNX4 increases ATG9A localization on autolysosomes upon starvation-induced autophagy

**Fig. 5.**
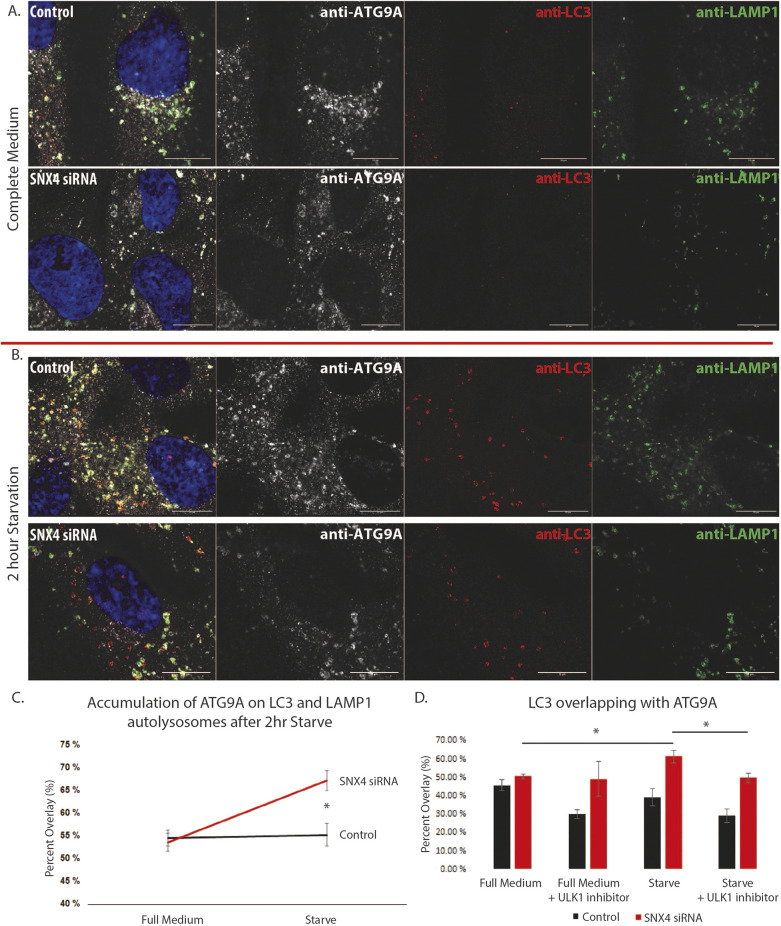
**In SNX4 depleted cells, ATG9A increases on LC3- and LAMP1-positive autolysosomes.** (A) Representative immunofluorescence micrographs showing ATG9A (greyscale) overlapping with both LC3 (red) and LAMP1 (green) in full medium. (B) Representative immunofluorescence micrograph showing ATG9A overlapping with both LC3 and LAMP1 after 2 h starvation. In A and B, cells were treated with scrambled siRNA (control) or siRNA targeting SNX4, as indicated. Left-hand panels show merge images, with DNA stained using Hoechst (blue). (C) Manders’ Overlap quantifications. Under starved conditions, ATG9A increases colocalization with autolysosomes upon SNX4 depletion. *n*=3 experiments. (D) Manders’ overlap quantifications of LC3 and ATG9A in full medium and starved conditions, with or without 1 μM ULK1 inhibitor. *n*=3 experiments. Mean±s.e.m. *P<0.05 (Student's *t*-test). Scale bars: 10 μm.

To assess whether the ATG9A increases on late endosomes and lysosomes are related to a decrease in autophagy, we investigated the presence of ATG9A on autolysosomes, characterized by the presence of both LC3 and LAMP1. In basal, full-medium conditions, we again observed the juxtanuclear ATG9A staining. In RPE-1 control cells, ATG9A was present on 54.5%±1.8 (mean±s.e.m.) of LC3-positive, LAMP1-positive autolysosome structures (Fig. 5A,C). Upon 2 h starvation in control cells, there was no increase of ATG9 on autolysosomes (55.2%±2.4; Fig. 5B,C). In SNX4-depleted cells, there was a marked decrease in the total number of LC3-positive structures, consistent with the findings in Fig. 4. Nevertheless, the relative colocalization of ATG9A with autolysosomes (LC3 and LAMP1-positive) increased upon amino acid starvation (full medium, 53.6%±2.0; starvation, 67.1%±2.2; Fig. 5A–C). This relative increase of ATG9A on autolysosomes in cells lacking SNX4 is presumably due to the inability of ATG9A to recycle from the autolysosomes for reutilization in another round of autophagy. Upon inhibition of autophagy with an inhibitor of the autophagy-inducing kinase ULK1, we observed a decrease of LC3 staining, as expected. Interestingly, however, the few LC3 puncta that could be observed in SNX4-depleted cells with ULK1 inhibition had a similar proportion of ATG9A colocalization as untreated cells (Fig. 5D; Fig. S1). This indicates that ATG9A does not depend on autophagy in order to be delivered to (auto)lysosomes.

In order to understand whether lysosomal and/or autolysosomal compartments had an altered ultrastructure upon SNX4 depletion, we labelled these organelles with internalized BSA–gold particles (Fig. S2). We did not observe any obvious changes in the ultrastructure of lysosomes and/or autolysosomes upon SNX4 depletion, but the total number of BSA–gold-containing vesicles increased upon starvation, probably due to the formation of gold-containing autolysosomes upon autophagy induction. Interestingly, in SNX4-depleted cells, the number of BSA–gold-containing vesicles was increased both under fed and starved conditions. While we do not know the reason for this, one possibility is that reduced recycling from lysosomes and autolysosomes causes their numerical expansion.

### SNX4 is required for proper autophagic flux

**Fig. 6.**
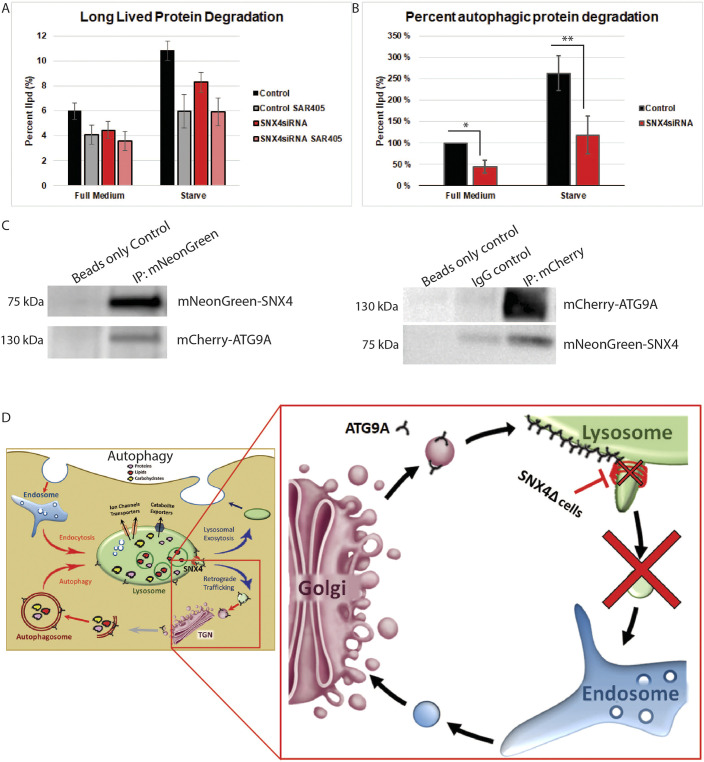
**SNX4 depletion prevents proper autophagic flux.** (A) Quantification of the percentage total degraded long-lived proteins (long-lived protein degradation, llpd) in cells with or without SNX4 siRNA and SAR405 treatments, as indicated, when in full medium or in starvation conditions. *n*=3 experiments normalized to percentage of control proteins degraded. Mean±s.e.m. (B) Quantification of the percent change in autophagic long-lived protein degradation by scrambled siRNA-treated (control) and SNX4 siRNA-treated cells in full medium or starvation conditions. *n*=3 experiments normalized to percentage of control proteins degraded. Mean±s.e.m. *P=0.049 and **P=0.002 for full medium and starved, respectively (Student's *t*-test). (C) Co-immunoprecipitation from RPE-1 cell lysates co-expressing mNeonGreen-SNX4 and mCherry-ATG9A using antibodies against the indicated fluorescent tags for immunoprecipitation (IP). (D) Graphical model of SNX4-dependent ATG9A recycling from autolysosomes to endosomes. TGN, trans-Golgi network.

To quantitatively assess the effect of SNX4 on bulk autophagic flux, we performed a long-lived protein degradation assay (Bauvy et al., 2009). This pulse-chase labelling approach showed that inhibiting SNX4 in amino acid-starved cells decreased the total protein degradation by 2.4%±0.2, 2.7%±0.2 and 2.5%±0.2 (mean±s.e.m.; three separate experiments) when compared to that in wild-type cells. Effectively, this showed that cells lacking SNX4 had a 44.4% reduction in protein degradation when compared to that of control cells in full medium (Fig. 6A) and a 44.9% reduction in protein degradation when compared to that of wild-type cells upon starvation (Fig. 6B). Taken together, these data show that SNX4 is required for proper autophagic flux.

To determine whether SNX4 interacts with ATG9A, we performed co-immunoprecipitation experiments. In cells co-expressing mNeonGreen–SNX4 and mCherry–ATG9A, we could detect mCherry–ATG9A in mNeonGreen immunoprecipitates and mNeonGreen–SNX4 in mCherry immunoprecipitates (Fig. 6C). However, when we performed a yeast two-hybrid assay to test possible direct interactions, we did not observe any interactions between SNX4 and the ATG9A N-terminus, ATG9A C-terminus nor with the full-length ATG9A (data not shown). This suggests that SNX4 and ATG9A could be part of the same multiprotein complex during endolysosomal sorting, without interacting directly.

## DISCUSSION

Although PI3P has a well-described function in protein recruitment to the growing phagophore, we have here uncovered a new role of this lipid in autophagy. We have used microscopy of live and fixed cells to show the requirement of the PI3P effector SNX4 for proper autophagic flux and for recycling of the lipid scramblase ATG9A from endolysosomes and autolysosomes. It has been observed that Snx4 can bind phosphatidylserine in budding yeast (Ma et al., 2018), but the punctate localization of Snx4 is dependent on the presence of PI3P (Hettema et al., 2003). Mammalian SNX4 is PI3P dependent, as observed with its disassociation from vesicles upon inhibition of the class III phosphatidylinositol 3-kinase VPS34 by SAR405. We propose that recycling of ATG9A from endolysosomes and autolysosomes sustains autophagy by allowing the same ATG9A molecule to be used for multiple cycles of autophagosome formation (Fig. 6D).

ATG9A vesicles are mobile, and their trafficking is controlled via nutrient-regulated signals (Young et al., 2006). Previous studies have found the importance of ATG9A during autophagosome formation, proposedly by functioning in vesicular delivery to the phagophore initiation site, and by translocating lipids from the outer to the inner phagophore membrane in order to enable its expansion (Maeda et al., 2020; Matoba et al., 2020; Orsi et al., 2012). Upon starvation-induced autophagy, ATG9A redistributes from the perinuclear Golgi regions to an enrichment juxtaposed to the autophagosome initiation site (Judith et al., 2019; Lamb et al., 2016; Longatti et al., 2012; Young et al., 2006). Because ATG9A has been shown to be involved in the early steps of autophagy, we suggest that recycling of ATG9A from the endolysosome back to the Golgi is required to sustain proper autophagy.

SNX proteins have been previously implicated in tubular sorting of endocytic proteins and are involved in endosome to Golgi retrograde transport of ricin (Skanland et al., 2007). Interestingly, binding of another PX–BAR domain-containing protein, SNX18, to phosphatidylinositol (4,5)-bisphosphate is required for regulation of autophagy via control of ATG9A trafficking from recycling endosomes and formation of ATG16L1- and WIPI2-positive autophagosome precursor membranes (Knaevelsrud et al., 2013). Thus, cycling of membrane proteins via SNX–BAR proteins is necessary for proper autophagy. In this study, we show a colocalization of SNX4 and ATG9A in several cellular compartments. When SNX4 is inhibited, ATG9A is unable to traffic correctly and remains in a more juxtanuclear Golgi zone. Although this is true in full medium conditions, it is even more pronounced during starvation-induced autophagy.

We found that, in RPE-1 cells lacking SNX4, proper autophagic responses are altered, as revealed by several independent autophagy assays, including fluorescence microscopy and long-lived protein degradation assays. During starvation-induced autophagy, there was a decrease in LC3 puncta and in long-lived protein degradation. Even though our data point to a role for SNX4 in ATG9A recycling, it is also conceivable that SNX4 regulates autophagy through additional mechanisms. Evidence from budding yeast suggests that Snx4 promotes autophagy and vacuole membrane fusion. Upon starvation-induced autophagy in cells lacking Snx4, phosphatidylserine accumulates in the membranes of the endosome and vacuole, autophagy intermediates accumulate within the cytoplasm, and homotypic vacuole fusion is impaired. The Snx4–Atg20 dimer displays a preference for binding and remodelling of phosphatidylserine-containing membrane *in vitro*, which suggests that Snx4–Atg20-coated carriers export phosphatidylserine-rich membrane from the endosome (Ma et al., 2018). Whether SNX4 is directly required for maintaining glycerophospholipid homeostasis and autophagy/vacuole fusion, remains to be investigated in mammalian cells.

Overall, the data herein provide a novel recycling process of integral membrane proteins from endolysosomes in mammalian cells, similar to protein recycling from the vacuole in budding yeast (Suzuki and Emr, 2018). During autophagy, SNX4 is recruited to endolysosomes via its PI3P-binding PX domain and is required for recycling of ATG9A. In the absence of SNX4, ATG9A and, presumably, additional unidentified proteins are unable to be recycled back to the Golgi for utilization in additional rounds of autophagy. Under stress, this inability to properly recycle ATG9A prevents proper autophagic flux from occurring. Overall, our findings indicate that PI3P is not only important for the initial stages of autophagy but also for the late stage of autophagy through recycling of ATG9A.

## MATERIALS AND METHODS

### Cell culture and generation of stable cell lines

hTERT-RPE-1 cells (human retinal pigment epithelial cells immortalized with telomerase) and stable cell lines derived from these cells were maintained in DMEM-F12/Dulbecco's Modified Eagle's Medium high glucose (DMEM, Sigma-Aldrich, D0819) supplemented with 10% fetal bovine serum (Sigma-Aldrich, F7524), 100 U/ml penicillin and 100 μg/ml streptomycin. All cells were cultured in a humidified incubator at 37°C supplemented with 5% CO_2_. For amino acid and growth factor starvation experiments, the growth medium was removed, cells were washed three times with EBSS (GIBCO BRL, 24,010–043) and replaced with EBSS or Live-Cell Imaging Solution (Molecular Probes, A14291DJ), supplemented with 20 mM glucose (Merck, 108,342).

Both stable cell lines used in these studies were lentivirus-generated pools, using plasmids pcDH-PGK-mNeonGreen-SNX4-IRES-Puro and pCDH-PGK-mCherry-ATG9A-IRES-Neo (described below). The weak PGK promoter was used for transgene expression at low expression levels. Third-generation lentivirus was generated as previously published (Campeau et al., 2009). Briefly, mCherry and mNeonGreen fusions were generated as Gateway ENTRY plasmids using standard molecular biology techniques. From these vectors, lentiviral transfer vectors were generated by recombination into customized pCDH (System Biosciences CD532-A) destination vectors using a Gateway LR reaction. VSV-G pseudotyped lentiviral particles were packaged using a third-generation packaging system (Addgene 12251, 12253 and 12259; deposited by Didier Tromo). Cells were then transduced with low virus titers, and stable expressing populations were generated by antibiotic selection.

### Antibodies

The following antibodies were used for the studies: hamster anti-ATG9A (Webber and Tooze, 2010; immunofluorescence, 1:500), rabbit anti-LAMP1 from Sigma-Aldrich (L1418; immunofluorescence, 1:200), mouse anti-LAMP1 from BD (555798; immunofluorescence, 1:500), human anti-EEA1 provided by Ban-Hock Toh (Monash University, Melbourne, Australia; immunofluorescence, 1:160,000), mouse anti-GM130 from BD (610823; immunofluorescence, 1:250) and sheep anti-TGN46 from Biorad (AHP500; immunofluorescence, 1:500). Hoechst 33342 was from Invitrogen Molecular Probes (H3570).

### Immunostaining

Cells grown on coverslips were fixed with 4% formaldehyde (Polyscience, 18814) for 20 min at room temperature and permeabilized with 0.05% saponin (Sigma-Aldrich, S7900) or 0.05% digitonin (Sigma-Aldrich, D141) in phosphate-buffered saline (PBS) for 5 min. Cells were then blocked in 5% BSA for 20 min and stained with the indicated primary antibody concentrations for 1 h in 1% BSA. The coverslips were then washed in PBS containing saponin for 5 min and stained for 1 h with fluorescently labelled secondary antibody at 1:1000 concentration in the dark. The cells were then washed with PBS and water and were mounted using mowiol (Sigma-Aldrich), either alone or supplemented with Hoechst 33342.

### siRNA transfections

Silencer Select siRNAs against human SNX4 and VPS35, and nontargeting control ‘scrambled’ siRNA (predesigned, 4390844) were purchased from Ambion (Thermo Fisher Scientific). Cells at 30% confluency were transfected with 20 nM final siRNA concentration using Lipofectamine RNAiMax transfection reagent (Life Technologies, 13778–150) according to the manufacturer's instructions and used for experiments after 48 h for VPS35 or 72 h for SNX4. All siRNA oligonucleotides have been validated previously for target specificity, and knockdown levels were routinely confirmed by western blotting or immunofluorescence imaging.

### Immunoblotting

Cells were washed in cold PBS and lysed in 2× Laemmli sample buffer (Bio-Rad Laboratories, 1610737) supplemented with dithiothreitol (DTT). Whole-cell lysates were separated by SDS–PAGE on 4–20% gradient gels (mini-PROTEAN TGX; Bio-Rad). Proteins were transferred to polyvinylidene difluoride (PVDF) membranes (TransBlot TurboTM LF PVDF, Bio-Rad) followed by 1 h blocking in 3% BSA and overnight antibody incubation in Tris-buffered saline with 0.1% Tween-20 (Sigma-Aldrich, P1379) at 4°C. Membranes incubated with HRP (horseradish peroxidase)-conjugated antibodies (HRP-conjugated anti-rabbit IgG, 111 035 144; HRP-conjugated anti-mouse IgG, 115 035 146; both Jackson ImmunoResearch) were developed using Clarity western ECL substrate solution (Bio-Rad) with a ChemiDoc XRS+ imaging system (Bio-Rad).

### Immunoprecipitation

Cells were collected and lysed in 400 μl NP40-Tris lysis buffer [50 mM Tris-HCl (pH 7.2), 125 mm potassium acetate, 2.5 mM magnesium acetate, 5 mM EGTA, 0.5% NP40 supplemented with Complete Mini EDTA-free protease inhibitor cocktail (Roche Applied Science)] on ice for 30 min, with mixing every 5 min. Nuclei and cell debris were cleared by centrifugation (16,000 ***g***, 15 min, 4°C). 50 μl protein G dynabeads (ThermoFisher, 10004D) were directly incubated with 1 μg of either anti-mNeonGreen monoclonal antibody (Chromotek, 32f6-20) or anti-mCherry antibody (Acris Antibodies, AB0040-200) and incubated with constant rotation for 10 min. The lysates were then added to the beads and immunoprecipitated at constant rotation for 30 min. The beads and associated proteins were washed three times using lysis buffer and then boiled in Laemmli buffer containing 100 mM DTT for 10 min to elute associated proteins. The eluted proteins were subjected to SDS–PAGE and detected by immunoblotting.

### Confocal fluorescence microscopy

Confocal images were obtained using an LSM 880 with Airyscan confocal microscope (Carl Zeiss) equipped with 405, 458, 488, 514, 561 and 633 nm laser lines, and 10× NA 0.45 DIC II (Plan-Apochromat), 20× NA 0.8 DIC II (Plan-Apochromat), 25× NA 0.8 (LD LCI Plan-Apochromat), 40× NA 1.2 Water Imm DIC III (C-Apochromat) and 63× NA 1.4 oil DIC III (Plan-Apochromat) objectives. Images were acquired using the 63×/1.40 oil DIC III objective.

### Electron microscopy

In order to label lysosomal compartments for ultrastructural and quantitative analysis by electron microscopy, we pulse-chased RPE cells with BSA–gold particles (Sigma-Aldrich, 520918-1G). Cells were incubated for 3 h with BSA–gold at 37°C, followed by a chase overnight. The next day, cells were washed three times with medium and then fixed in 2% glutaraldehyde in 0.1 M PHEM buffer (60 mM PIPES, 25 mM HEPES, 2 mM MgCl_2_, 10 mM EGTA, pH 6.9) for 2 h. Postfixation was 1 h with 2% OsO_4_ and 1.5% K_4_(Fe(CN)_6_) in 0.1 M PHEM, followed by 0.5% tannic acid (30 min). After staining with 4% uranyl acetate in distilled H_2_O (30 min), the coverslips were dehydrated in a graded series of ethanol and embedded in Epon (Sigma-Aldrich, 45359-1EA-F). For morphological analysis and quantification, we prepared 100 nm sections on a Leica utramicrotome and observed these in a JEOL-JEM 1230 at 80 kV. For quantification, we counted the total number of gold-containing vesicles on ten individual cell sections in two separate experiments.

### Image processing and data analysis

hTERT-RPE-1 cells stably expressing mNeonGreen–SNX4 were fixed and stained with different antibodies. Images were acquired by concocal fluorescence microscopy at fixed intensity below saturation. Colocalization was then quantified with FIJI (https://imagej.net/Fiji) using the JACoP plugin (Bolte and Cordelieres, 2006). Manders’ Overlap Coefficient was used to describe the amount of overlap (Dunn et al., 2011).

### Long-lived protein degradation assay

Cells were incubated for 36 h with 0.135 μCi/ml L-[^14^C]valine supplemented complete RPMI (with 10% FBS and 1% penicillin and streptomycin) medium, followed by two washes and a 3 h chase for degradation of short-lived proteins in fresh medium containing 10 mM nonradioactive L-valine. Next, cells were washed and treated with either EBSS (starvation) or complete RPMI medium with and without SAR405 (3 μM; Selleckchem, S7682) for 4 h. For each sample, two measurements of radioactivity were made, the acid-soluble fraction of the medium and the acid-insoluble fraction of the medium together with the cells remaining in the well. Radioactivity was then measured using a scintillation analyser, counting for 3 min/sample. Percent degradation was defined as the acid-soluble radioactivity released into the medium divided by the total radioactivity. Percent degradation from autophagy was calculated by comparing the SAR405-treated versus untreated samples.

### Statistical analysis

Statistical analysis was performed using Graphpad Prism. Student's two-tailed *t*-test was used to test for statistical significance in samples with a Gaussian distribution. In order to account for differences in staining efficiencies and imaging conditions, experiments involving quantification of intensities were normalized by the mean of the experiment and then analysed. This is also true of the percentage long-lived protein degradation experiments.

## Supplementary Material

Supplementary information

Reviewer comments
