# Supplementary material for: The phosphatidylinositol 3-phosphate-binding protein SNX4 controls ATG9A recycling and autophagy
Source: J Cell Sci. 2021 Feb 10;134(3):jcs250670. doi: 10.1242/jcs.250670 (PMC7888711; doi:10.1242/jcs.250670)
Supplement: Supplementary information [file joces-134-250670-s1.pdf]

SUPPLEMENTARY INFORMATION

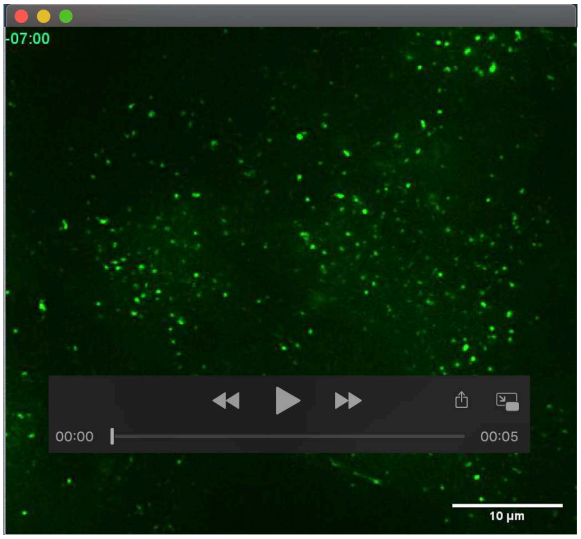

Movie 1. Association of mNeonGreen-SNX4 with endolysosomes depends on PI3P.

Time scale is in minutes where the class III phosphatidylinositol 3-kinase VPS34 inhibitor SAR405 was added at time point 0.

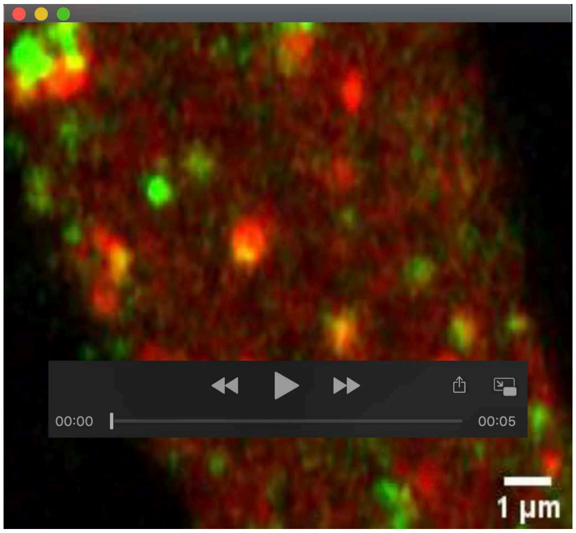

Movie 2. Live cell microscopy showing the relationship of ATG9A with SNX4 in nGreen-SNX4 mCherry-ATG9A stable cell lines.

ATG9A is marked in red and SNX4 in green.

Journal of Cell Science • Supplementary information

Supplementary Figure 1

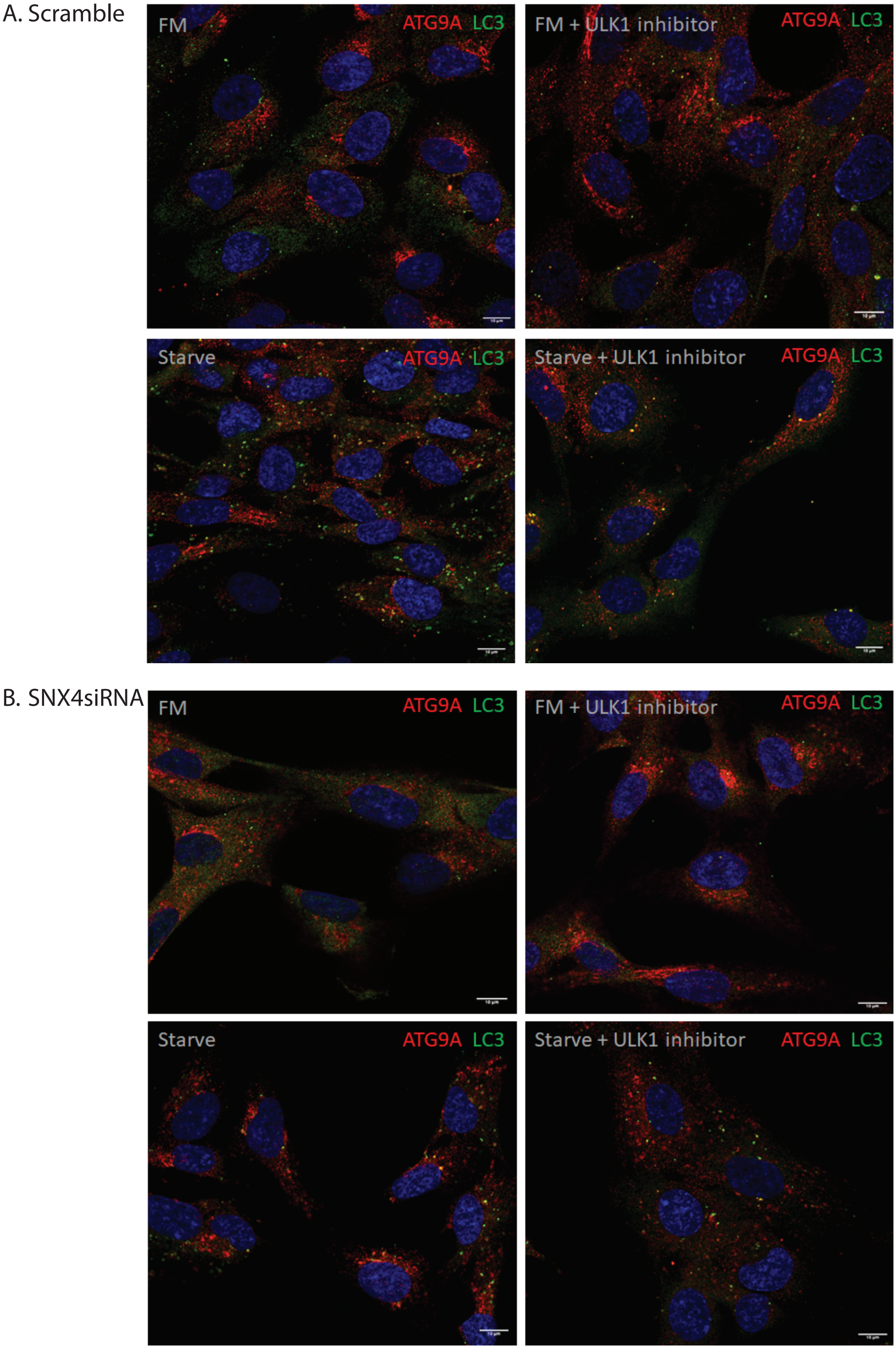

Figure S1. Representative immunofluorescence micrograph of LC3 and ATG9A in scramble control (upper panel) and SNX4 depleted (lower panel) cells with or without ULK1 inhibitor in full medium or starved condition

Supplementary Figure 2

A.

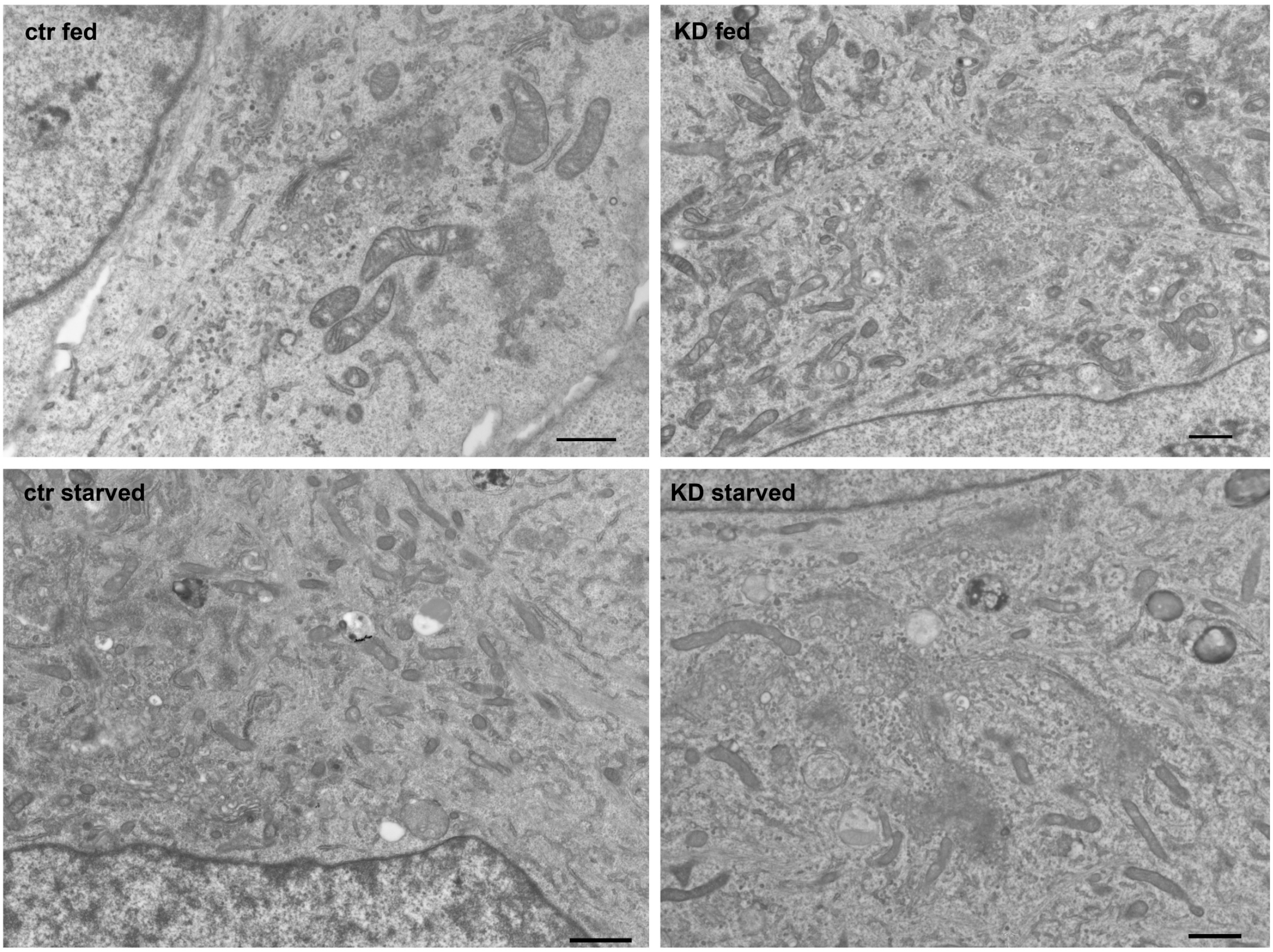

B.

Control Full Medium	Control Starved	SNX4siRNA Full Medium	SNX4siRNA Starved
2.45 ± 0.21	3.35 ± 1.34	4.85 ± 1.20	9.25 ± 1.48

Figure S2. Ultrastructure of cells in control and SNX4-depleted cells in full medium and starved conditions

A. Transmission electron microscopy showed no obvious ultrastructural differences in fed or starved conditions between control cells vs. SNX4 depleted cells.
B. Quantifications of gold containing lysosomes/autolysosomes upon 2 hour starvation in control vs. SNX4-depleted cells.
