# Supplementary material for: The phosphatidylinositol 3-phosphate-binding protein SNX4 controls ATG9A recycling and autophagy
Source: J Cell Sci. 2021 Feb 10;134(3):jcs250670. doi: 10.1242/jcs.250670 (PMC7888711; doi:10.1242/jcs.250670)
Supplement: Reviewer comments [file joces-134-250670_review_history.pdf]

Journal of Cell Science

The phosphatidylinositol 3-phosphate-binding protein SNX4 controls ATG9A recycling and autophagy

Anthony Ravussin, Andreas Brech, Sharon A. Tooze and Harald Stenmark
DOI: 10.1242/jcs.250670

Editor: Tamotsu Yoshimori

Review timeline
Original submission: 24 June 2020
Editorial decision: 20 July 2020
First revision received: 24 November 2020
Accepted: 21 December 2020

Original submission

First decision letter

MS ID#: JOCES/2020/250670

MS TITLE: The phosphatidylinositol 3-phosphate binding protein SNX4 controls ATG9A recycling and autophagy

AUTHORS: Harald Stenmark, Anthony Ravussin, and Sharon Tooze
ARTICLE TYPE: Research Article

We have now reached a decision on the above manuscript.

To see the reviewers' reports and a copy of this decision letter, please go to: https://submit-jcs.biologists.org and click on the 'Manuscripts with Decisions' queue in the Author Area. (Corresponding author only has access to reviews.)

As you will see, although both reviewers felt that your findings, in principle, would be of interest to our JCS readership, they raise several substantial criticisms that prevent me from accepting the paper at this stage. We think, however, that a revised version might prove acceptable, if you can address their concerns. If you think that you can deal satisfactorily with the criticisms on revision, I would be pleased to see a revised manuscript. We would then return it to the reviewers.

We are aware that you may currently be unable to access the lab to undertake experimental revisions. If it would be helpful, we encourage you to contact us to discuss your revision in greater detail. Please send us a point-by-point response indicating where you are able to address concerns raised (either experimentally or by changes to the text) and where you will not be able to do so within the normal timeframe of a revision. We will then provide further guidance. Please also note that we are happy to extend revision timeframes as necessary.

Please ensure that you clearly highlight all changes made in the revised manuscript. Please avoid using 'Tracked changes' in Word files as these are lost in PDF conversion.

I should be grateful if you would also provide a point-by-point response detailing how you have dealt with the points raised by the reviewers in the 'Response to Reviewers' box. Please attend to all of the reviewers' comments. If you do not agree with any of their criticisms or suggestions please explain clearly why this is so.

© 2020. Published by The Company of Biologists under the terms of the Creative Commons Attribution License (https://creativecommons.org/licenses/by/4.0/).

Reviewer 1

Advance summary and potential significance to field

This manuscript entitled “The phosphatidylinositol 3-phosphate binding protein SNX4 controls ATG9A recycling and autophagy” by Anthony Ravussin et. al. revealed critical role of phosphatidylinositol 3-phosphate (PI3P) binding SNX4 protein in autophagy. SNX4 mediates recycling of the ATG9A from endolysosomes to early endosomes. Upon knock down of ANX4, autophagy flux is abrogated, and ATG9A becomes accumulated at autolysosomes. ATG9A is proposed to retrieved to endosome and Golgi from autolysosomes. The data are mostly clean and convincing. It will merit to the readers to understand how ATG9A is transported within a cell.

Comments for the author

One major concern about the authors’ model is that the route through which ATG9A is delivered to autolysosome is ambiguous. The authors claimed that autophagy mediate the delivery, however it seems inconsistent, since SNX4 knock down will abrogate autophagy while ATG9A is much more accumulated in endolysosmes even in such condition. This point should be clarified before acceptance.

Major point 1. As mentioned above, the authors need to experimentally show which pathway is employed to deliver ATG9A to endolysosome. One possibility is that SNX4 knock down did not completely block autophagy and residual autophagy activity may be sufficient for the delivery. The authors need to robustly knock down or knock out ATG gene(s) together with SNX4 to completely block autophagy, and see if ATG9A is still accumulated in endolysosome under starvation.

Reviewer 2

Advance summary and potential significance to field

In this manuscript, author described the role of SNX4 in recycling of ATG9A, furthermore, they demonstrate that the recycling of ATG9A by SNX4 is required for sustain autophagy. Beside find SNX4 mediate ATG9A recycling, the concept of recycling of ATG9A from autolysosome to sustain autophagy is interesting.

Comments for the author

1)Is that possible to directly visualize the recycling of ATG9A from autolysosome by living cell imaging?
2)Does SNX4 interact with ATG9A?
3) It will be nice to include TEM images of SNX4 depleted cells, control and starved.
4)Are there any change in LC3 lipidation level in SNX4 depleted cells?

First revision

Author response to reviewers' comments

Reviewer 1

This manuscript entitled “The phosphatidylinositol 3-phosphate binding protein SNX4 controls ATG9A recycling and autophagy” by Anthony Ravussin et. al. revealed critical role of phosphatidylinositol 3-phosphate (PI3P) binding SNX4 protein in autophagy. SNX4 mediates recycling of the ATG9A from endolysosomes to early endosomes. Upon knock down of ANX4, autophagy flux is abrogated, and ATG9A becomes accumulated at autolysosomes. ATG9A is proposed to retrieved to endosome and Golgi from autolysosomes. The data are mostly clean and convincing. It will merit to the readers to understand how ATG9A is transported within a cell.

© 2020. Published by The Company of Biologists under the terms of the Creative Commons Attribution License (https://creativecommons.org/licenses/by/4.0/).

We thank the reviewer for positive comments, and also for providing very helpful suggestions for improvement of our manuscript.

One major concern about the authors' model is that the route through which ATG9A is delivered to autolysosome is ambiguous. The authors claimed that autophagy mediate the delivery, however it seems inconsistent, since SNX4 knock down will abrogate autophagy, while ATG9A is much more accumulated in endolysosmes even in such condition. This point should be clarified before acceptance.

Major point:
As mentioned above, the authors need to experimentally show which pathway is employed to deliver ATG9A to endolysosome. One possibility is that SNX4 knock down did not completely block autophagy and residual autophagy activity may be sufficient for the delivery. The authors need to robustly knock down or knock out ATG gene(s) together with SNX4 to completely block autophagy, and see if ATG9A is still accumulated in endolysosome under starvation.

The reviewer makes a valid point, although the scope of our study was not to explain how ATG9A is delivered to endolysosomes but rather how it is recycled from lysosomes and endolysosomes. To address whether autophagy inhibition as such affects trafficking of ATG9A to endolysosomes, we used an inhibitor of ULK1, which potently inhibits autophagy. Firstly, we recapitulated the phenotype that SNX4 knockdowns led to decreased LC3 puncta upon starvation. In contrast, the fraction of LC3 vesicles containing ATG9A increased in SNX4 depleted cells. As expected, when we used ULK1 inhibition, we observed a strong decrease of LC3 puncta. Interestingly, however, a few LC3 puncta could be detected even in ULK1-inhibited cells, and SNX4-depleted cells with ULK1 inhibition had normal proportion of LC3 vesicles containing ATG9A. This indicates that ATG9A does not depend on autophagy in order to be delivered to the lysosomes that form autolysosomes. These data are included as new Figure 5D and new Supplementary Figure 1.

Reviewer 2

In this manuscript, author described the role of SNX4 in recycling of ATG9A, furthermore, they demonstrate that the recycling of ATG9A by SNX4 is required for sustain autophagy. Beside find SNX4 mediate ATG9A recycling, the concept of recycling of ATG9A from autolysosome to sustain autophagy is interesting.

We thank the reviewer for finding our work interesting, and for very good suggestions which have led to a further improvement of our manuscript.

1. Is that possible to directly visualize the recycling of ATG9A from autolysosome by living cell imaging?

ATG9A is a very highly dynamic protein, which is located throughout the cell. The trafficking of ATG9A occurs extremely fast with high mobility in small vesicles. Trying to identify co-localization with autolysosomes has been proven very difficult. Due to the resolution limitations of our microscopes, we were unable to visualize with certainty individual ATG9A particles recycling from autolysosomes.

2. Does SNX4 interact with ATG9A?

In cells co-expressing mNg-SNX4 and mCh-ATG9A, we could detect mCh-ATG9A in mNg immunoprecipitates and mNg-SNX4 in mCh immunoprecipitates. However, when we performed a yeast-2-hybrid assay to test possible direct interactions, we did not observe any interactions of SNX4 with neither ATG9A-N terminus, ATG9A-C terminus nor full- length ATG9A. We therefore think that SNX4 and ATG9A may be part of the same multi-protein (sorting) complex on endosomes without interacting directly. The co-immunoprecipitation experiments are included as new Figure 6C.

3. It will be nice to include TEM images of SNX4 depleted cells, control and starved.

As suggested, we performed the transmission electron microscopy of control and SNX4-depleted cells (new Supplementary Figure 2). There did not seem to be any overt ultrastructural differences

© 2020. Published by The Company of Biologists under the terms of the Creative Commons Attribution License (https://creativecommons.org/licenses/by/4.0/).

between the control cells vs SNX4 depleted cells, neither full medium nor after 2 hour starvation conditions. The electron microscopy was performed by prof. Andreas Brech, who is now included as co-author.

4. Are there any change in LC3 lipidation level in SNX4 depleted cells?

We investigated by Western blotting whether SNX4 depletion led to changes in LC3 lipidation and could indeed detect differences in LC3 lipidation levels (new Figure 4D). Interestingly, in full medium, SNX4 depleted cells had a higher proportion of LC3-II to LC3-I than control cells. This would suggest that there is a higher level of baseline LC3-II lipidation in SNX4-depleted cells. This could be due to the decreased turnover of LC3-II, in agreement with our finding of lower autophagic flux in SNX4 depleted cells. This phenotype seems quite similar to cells treated with protease inhibitor, E64d, where there is more LC3-II levels in nutrient-rich conditions when compared to untreated wild type cells. (Ref. Tanida et al., below). Upon starvation, however, we did not observe any significant differences in relative LC3-II levels in SNX4-depleted cells vs control cells. This also fits with our fluorescence microscopy data.

Reference:
Tanida I., Ueno T., Kominami E. (2008) LC3 and Autophagy. In: Deretic V. (eds) Autophagosome and Phagosome. Methods in Molecular Biology™, vol 445. Humana Press. https://doi.org/10.1007/978-1-59745-157-4_4

Second decision letter

MS ID#: JOCES/2020/250670

MS TITLE: The phosphatidylinositol 3-phosphate binding protein SNX4 controls ATG9A recycling and autophagy

AUTHORS: Harald Stenmark, Andreas Brech, Anthony Ravussin, and Sharon Tooze
ARTICLE TYPE: Research Article

I am happy to tell you that your manuscript has been accepted for publication in Journal of Cell Science, pending standard ethics checks.

Reviewer 1

Advance summary and potential significance to field

The authors properly responded to my concerns.

Comments for the author

The result is clear and now suitable for publication.

Reviewer 2

Advance summary and potential significance to field

In this manuscript, author described the role of SNX4 in recycling of ATG9A, furthermore, they demonstrate that the recycling of ATG9A by SNX4 is required for sustain autophagy. Beside find SNX4 mediate ATG9A recycling, the concept of recycling of ATG9A from autolysosome to sustain autophagy is interesting.

© 2020. Published by The Company of Biologists under the terms of the Creative Commons Attribution License (https://creativecommons.org/licenses/by/4.0/).

Comments for the author

Authors had addressed my queries satisfactorily, congratulations for this interesting work.

© 2020. Published by The Company of Biologists under the terms of the Creative Commons Attribution License (https://creativecommons.org/licenses/by/4.0/).
